# Ethyl 4-oxo-1,4-di­hydro­pyridine-3-carboxyl­ate

**DOI:** 10.1107/S2414314621005551

**Published:** 2021-06-04

**Authors:** Jun Gao, Sihui Long

**Affiliations:** a School of Chemical Engineering and Pharmacy, Wuhan Institute of Technology, Wuhan, Hubei 430205, People’s Republic of China; University of Kentucky, USA

**Keywords:** crystal structure, bifurcated hydrogen bonds

## Abstract

The title compound, C_8_H_9_NO_3_, likely generated through hydrolysis and esterification of 3′-carb­oxy-3-methyl-(1,4′-bipyridin)-1-ium chloride by ethanol, which contained water, has a nearly planar conformation. The crystal structure is sustained by one-dimensional chains along the *a*-axis direction based on bifurcated N—H⋯(O,O) hydrogen bonds between the NH group of the 4-oxo-1,4-di­hydro­pyridine ring and the two carbonyl O atoms.

## Abstract 2

Crystal growth of 3′-carb­oxy-3-methyl-[1,4′-bipyridin]-1-ium chloride in ethanol led to single crystals of ethyl 4-oxo-1,4-di­hydro­pyridine-3-carb­oxy­lic acid, which likely resulted from hydrolysis and esterification of the original compound, and its crystal structure was determined by single-crystal X-ray diffraction.

## Graphical abstract

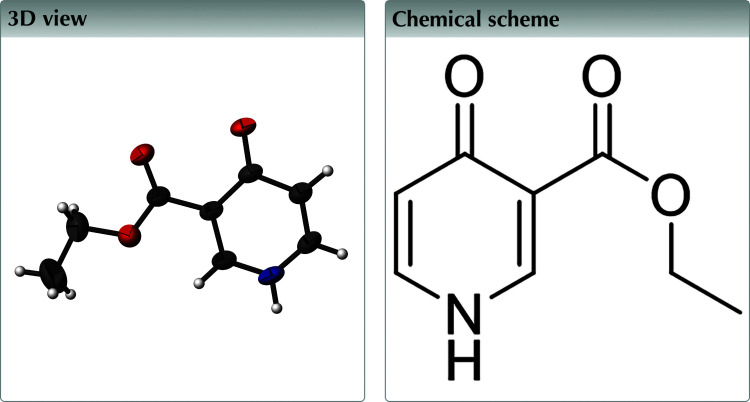

## Structure description

**Figure 1:**
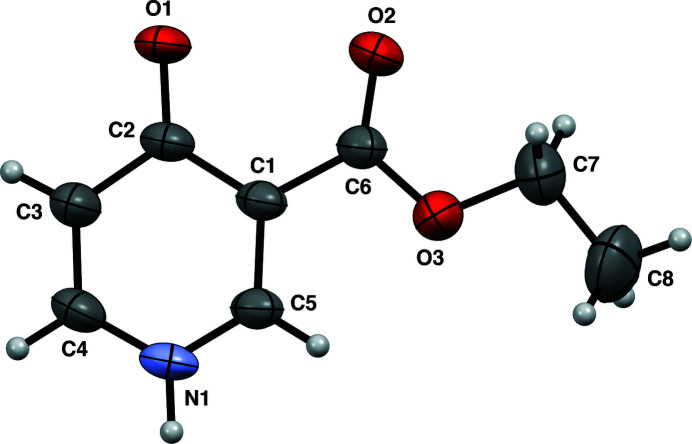
The mol­ecular structure of the title compound with displacement ellipsoids drawn at the 50% probability level.

**Figure 2:**
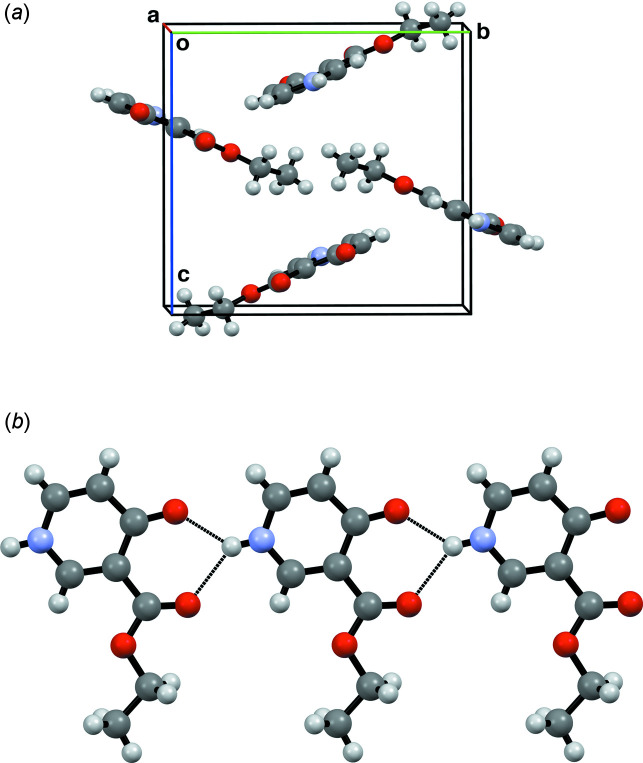
(*a*) Packing of the mol­ecules in the title compound viewed along the *a* axis; (*b*) Chain sustained by bifurcated hydrogen bonds between the NH group and two carbonyl O atoms (blue dashed lines).

The title compound (Fig. 1 ▸) was first synthesized by Ross (1966 ▸). It may be a potential inhibitor of the glycolytic process by which many cancer cells derive an appreciable proportion of their energy requirement (Ross, 1966 ▸). Balogh *et al.* (1980 ▸) demonstrated that the compound exhibited anti­microbial activity. In our study, the compound was obtained serendipitously during an attempt to grow single crystals of 3′-carb­oxy-3-methyl-(1,4′-bipyridin)-1-ium chloride in ethanol. The compound has a nearly planar conformation, as evidenced by the dihedral angle between the 4-oxo-1,4-di­hydro­pyridine ring and the ester moiety [2.3 (2)°]. In the crystal, the mol­ecules form chains propagating parallel to the *a*-axis through bifurcated hydrogen bonds between the NH group and the two carbonyl oxygen atoms. The hydrogen bond parameters for NH⋯O=C (ring) are: 1.96 (2) Å for bond length, and 134.9 (17)° for the bond angle. The corresponding parameters for NH⋯O=C (ester) are 2.15 (2) Å and 139.6 (17)° (Fig. 2 ▸, Table 1 ▸).

## Synthesis and crystallization

**Figure 3:**
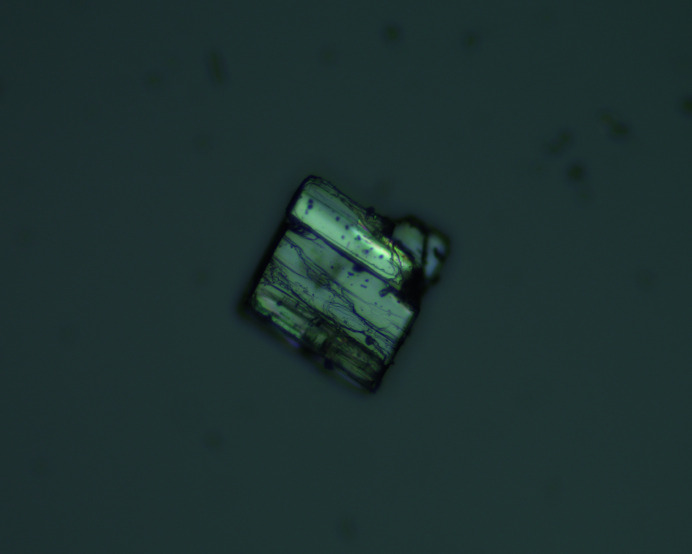
A representative crystal of I.

**Figure 4:**
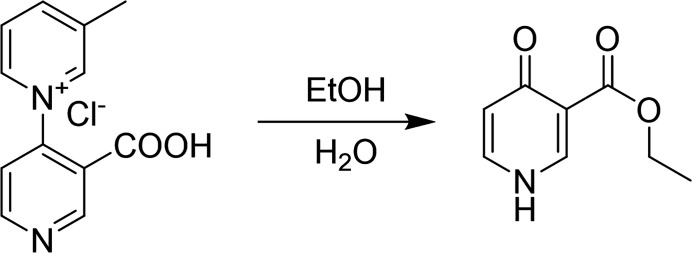
Reaction scheme.

The title compound was obtained during an attempt to grow single crystals of 3′-carb­oxy-3-methyl-(1,4′-bipyridin)-1-ium chloride by slow evaporation of an ethano­lic solution. 3′-Carb­oxy-3-methyl-(1,4′-bipyridin)-1-ium chloride was dissolved in bulk ethanol at 343 K, and then the resulting solution was left in a refrigerator. Colorless plate-shaped crystals (Fig. 3 ▸) were harvested after several days. Structure determination by single-crystal X-ray diffraction revealed the identity of the crystals to be ethyl 4-oxo-1,4-di­hydro­pyridine-3-carboxyl­ate. Hydrolysis and esterification of 3′-carb­oxy-3-methyl-[1,4′-bipyridin]-1-ium chloride may have led to the title compound (Fig. 4 ▸).

## Refinement

Crystal data, data collection and structure refinement details are summarized in Table 2 ▸.

## Supplementary Material

Crystal structure: contains datablock(s) global, I. DOI: 10.1107/S2414314621005551/pk4034sup1.cif

Structure factors: contains datablock(s) I. DOI: 10.1107/S2414314621005551/pk4034Isup2.hkl

Supporting information file. DOI: 10.1107/S2414314621005551/pk4034Isup3.cml

CCDC reference: 2086773

Additional supporting information:  crystallographic information; 3D view; checkCIF report

## Figures and Tables

**Table 1:** Hydrogen-bond geometry (Å, °)

*D*—H⋯*A*	*D*—H	H⋯*A*	*D*⋯*A*	*D*—H⋯*A*
N1—H1⋯O1^i^	0.90 (2)	1.96 (2)	2.6771 (15)	134.9 (17)
N1—H1⋯O2^i^	0.90 (2)	2.15 (2)	2.9002 (17)	139.6 (17)

Symmetry code: (i) 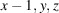 .

**Table 2:** Experimental details

Crystal data	
Chemical formula	C_8_H_9_NO_3_
*M*_r_	167.16
Crystal system, space group	Monoclinic, *P*2_1_/*c*
Temperature (K)	293
*a*, *b*, *c* (Å)	6.4973 (2), 11.5323 (5), 11.2908 (5)
β (°)	91.500 (4)
*V* (Å^3^)	845.72 (6)
*Z*	4
Radiation type	Cu *K*α
μ (mm^−1^)	0.86
Crystal size (mm)	0.07 × 0.03 × 0.02

Data collection	
Diffractometer	Rigaku Oxford Diffraction, Synergy Custom system, HyPix
Absorption correction	Multi-scan (*CrysAlis PRO*; Rigaku OD, 2020 ▸)
*T*_min_, *T*_max_	0.311, 1.000
No. of measured, independent and observed [*I* > 2σ(*I*)] reflections	5379, 1693, 1456
*R*_int_	0.022
(sin θ/λ)_max_ (Å^−1^)	0.633

Refinement	
*R*[*F* ^2^ > 2σ(*F* ^2^)], *wR*(*F* ^2^), *S*	0.046, 0.129, 1.11
No. of reflections	1693
No. of parameters	114
H-atom treatment	H atoms treated by a mixture of independent and constrained refinement
Δρ_max_, Δρ_min_ (e Å^−3^)	0.21, −0.22

Computer programs: *CrysAlis PRO* (Rigaku OD, 2020 ▸), *SHELXS* (Sheldrick, 2008 ▸), *SHELXL* (Sheldrick, 2015 ▸), *Mercury* (Macrae *et al.*, 2020 ▸) and *OLEX2* (Dolomanov *et al.*, 2009 ▸).

## full crystallographic data

### Crystal data

**Table:**

C_8_H_9_NO_3_	*F*(000) = 352
*M**_r_* = 167.16	*D*_x_ = 1.313 Mg m^−^^3^
Monoclinic, *P*2_1_/*c*	Cu *K*α radiation, λ = 1.54184 Å
*a* = 6.4973 (2) Å	Cell parameters from 3630 reflections
*b* = 11.5323 (5) Å	θ = 6.8–76.4°
*c* = 11.2908 (5) Å	µ = 0.86 mm^−^^1^
β = 91.500 (4)°	*T* = 293 K
*V* = 845.72 (6) Å^3^	Needle, clear light colourless
*Z* = 4	0.07 × 0.03 × 0.02 mm

### Data collection

**Table:**

Rigaku Oxford Diffraction, Synergy Custom system, HyPix diffractometer	1693 independent reflections
Radiation source: Rotating-anode X-ray tube, Rigaku (Cu) X-ray Source	1456 reflections with *I* > 2σ(*I*)
Mirror monochromator	*R*_int_ = 0.022
Detector resolution: 10.0000 pixels mm^-1^	θ_max_ = 77.4°, θ_min_ = 6.8°
ω scans	*h* = −8→7
Absorption correction: multi-scan (CrysAlisPro; Rigaku OD, 2020)	*k* = −14→5
*T*_min_ = 0.311, *T*_max_ = 1.000	*l* = −13→14
5379 measured reflections	

### Refinement

**Table:**

Refinement on *F*^2^	Primary atom site location: structure-invariant direct methods
Least-squares matrix: full	Hydrogen site location: mixed
*R*[*F*^2^ > 2σ(*F*^2^)] = 0.046	H atoms treated by a mixture of independent and constrained refinement
*wR*(*F*^2^) = 0.129	*w* = 1/[σ^2^(*F*_o_^2^) + (0.0674*P*)^2^ + 0.1472*P*] where *P* = (*F*_o_^2^ + 2*F*_c_^2^)/3
*S* = 1.11	(Δ/σ)_max_ < 0.001
1693 reflections	Δρ_max_ = 0.21 e Å^−^^3^
114 parameters	Δρ_min_ = −0.22 e Å^−^^3^
0 restraints	

### Special details

**Table:**

Geometry. All esds (except the esd in the dihedral angle between two l.s. planes) are estimated using the full covariance matrix. The cell esds are taken into account individually in the estimation of esds in distances, angles and torsion angles; correlations between esds in cell parameters are only used when they are defined by crystal symmetry. An approximate (isotropic) treatment of cell esds is used for estimating esds involving l.s. planes.

### Fractional atomic coordinates and isotropic or equivalent isotropic displacement parameters (Å^2^)

**Table:**

	*x*	*y*	*z*	*U*_iso_*/*U*_eq_	
O1	0.52166 (14)	0.40585 (10)	0.20366 (11)	0.0600 (4)	
O2	0.57531 (15)	0.62403 (10)	0.09977 (11)	0.0584 (4)	
O3	0.28325 (16)	0.71651 (9)	0.05152 (11)	0.0555 (3)	
N1	−0.08871 (17)	0.47182 (12)	0.17157 (11)	0.0471 (3)	
C1	0.25305 (18)	0.53513 (12)	0.14051 (12)	0.0388 (3)	
C2	0.33512 (19)	0.42840 (13)	0.19022 (13)	0.0436 (4)	
C3	0.1821 (2)	0.34638 (14)	0.22568 (15)	0.0538 (4)	
H3	0.224295	0.274979	0.255937	0.065*	
C4	−0.0203 (2)	0.36993 (15)	0.21635 (15)	0.0517 (4)	
H4	−0.114786	0.315080	0.241186	0.062*	
C5	0.04315 (19)	0.55121 (13)	0.13385 (13)	0.0421 (3)	
H5	−0.008399	0.619967	0.101877	0.051*	
C6	0.3901 (2)	0.62675 (12)	0.09660 (13)	0.0421 (3)	
C7	0.3996 (3)	0.81144 (16)	0.00264 (19)	0.0663 (5)	
H7A	0.470155	0.786241	−0.067519	0.080*	
H7B	0.501453	0.839160	0.060290	0.080*	
C8	0.2495 (4)	0.90531 (19)	−0.0281 (2)	0.0899 (7)	
H8A	0.317924	0.965688	−0.070384	0.135*	
H8B	0.194250	0.936516	0.043203	0.135*	
H8C	0.139659	0.874119	−0.076909	0.135*	
H1	−0.224 (3)	0.4880 (17)	0.1645 (17)	0.071 (6)*	

### Atomic displacement parameters (Å^2^)

**Table:**

	*U*^11^	*U*^22^	*U*^33^	*U*^12^	*U*^13^	*U*^23^
O1	0.0234 (5)	0.0609 (7)	0.0956 (9)	0.0027 (4)	0.0007 (5)	0.0191 (6)
O2	0.0296 (5)	0.0541 (7)	0.0918 (9)	−0.0041 (4)	0.0038 (5)	0.0110 (6)
O3	0.0411 (6)	0.0456 (6)	0.0799 (8)	0.0017 (4)	0.0029 (5)	0.0130 (5)
N1	0.0211 (5)	0.0596 (8)	0.0608 (7)	−0.0002 (5)	0.0018 (5)	0.0002 (6)
C1	0.0255 (6)	0.0448 (8)	0.0460 (7)	0.0000 (5)	0.0022 (5)	−0.0028 (6)
C2	0.0241 (6)	0.0512 (8)	0.0555 (8)	−0.0001 (5)	0.0018 (5)	0.0018 (6)
C3	0.0321 (7)	0.0519 (9)	0.0774 (11)	−0.0012 (6)	0.0020 (7)	0.0147 (8)
C4	0.0288 (7)	0.0588 (9)	0.0678 (10)	−0.0075 (6)	0.0045 (6)	0.0075 (7)
C5	0.0279 (6)	0.0467 (8)	0.0517 (8)	0.0028 (5)	0.0006 (5)	−0.0024 (6)
C6	0.0307 (6)	0.0423 (7)	0.0532 (8)	0.0010 (5)	0.0016 (5)	−0.0022 (6)
C7	0.0674 (11)	0.0478 (9)	0.0842 (12)	−0.0088 (8)	0.0079 (9)	0.0110 (8)
C8	0.1063 (19)	0.0616 (13)	0.1014 (17)	0.0040 (11)	−0.0053 (14)	0.0288 (11)

### Geometric parameters (Å, º)

**Table:**

O1—C2	1.2450 (16)	C1—C2	1.449 (2)
O2—C6	1.2032 (16)	C1—C5	1.3765 (17)
O3—C6	1.3395 (17)	C1—C6	1.4760 (19)
O3—C7	1.448 (2)	C2—C3	1.437 (2)
N1—C4	1.350 (2)	C3—C4	1.344 (2)
N1—C5	1.3314 (19)	C7—C8	1.492 (3)
			
C6—O3—C7	117.28 (12)	C4—C3—C2	121.85 (15)
C5—N1—C4	120.66 (12)	C3—C4—N1	121.15 (14)
C2—C1—C6	121.28 (11)	N1—C5—C1	122.33 (13)
C5—C1—C2	119.33 (12)	O2—C6—O3	122.66 (13)
C5—C1—C6	119.40 (12)	O2—C6—C1	125.66 (13)
O1—C2—C1	124.89 (13)	O3—C6—C1	111.68 (11)
O1—C2—C3	120.46 (14)	O3—C7—C8	107.00 (16)
C3—C2—C1	114.65 (12)		

### Hydrogen-bond geometry (Å, º)

**Table:**

*D*—H···*A*	*D*—H	H···*A*	*D*···*A*	*D*—H···*A*
N1—H1···O1^i^	0.90 (2)	1.96 (2)	2.6771 (15)	134.9 (17)
N1—H1···O2^i^	0.90 (2)	2.15 (2)	2.9002 (17)	139.6 (17)

Symmetry code: (i) *x*−1, *y*, *z*.
